# Assessing the impact of biochar and nitrogen application on yield, water-nitrogen use efficiency and quality of intercropped maize and soybean

**DOI:** 10.3389/fpls.2023.1171547

**Published:** 2023-05-08

**Authors:** Lixue Wang, Binhang Yu, Jianmei Ji, Ismail Khan, Guanlin Li, Abdul Rehman, Dan Liu, Sheng Li

**Affiliations:** ^1^ College of Water Conservancy, Shenyang Agricultural University, Shenyang, China; ^2^ School of the Environment and Safety Engineering, Jiangsu University, Zhenjiang, China; ^3^ Department of Agronomy, Faculty of Agriculture and Environment, The Islamia University of Bahawalpur, Bahawalpur, Pakistan

**Keywords:** maize-soybean intercropping, biochar, nitrogen application, yield, NRE, NUE

## Abstract

**Introduction:**

Biochar (BC) and nitrogen (N) application have the potential to increase grain yield and resource use efficiency in intercropping systems. However, the effects of different levels of BC and N application in these systems remain unclear. To address this gap, the study is intended to ascertain the impact of various combinations of BC and N fertilizer on the performance of maize-soybean intercropping and determine the optimum application of BC and N for maximizing the effect of the intercropping system.

**Methods:**

A two-year (2021-2022) field experiment was conducted in Northeast China to assess the impact of BC (0, 15, and 30 t ha^-1^) and N application (135, 180, and 225 kg ha^-1^) on plant growth, yield, water use efficiency (WUE), N recovery efficiency (NRE) and quality in an intercropping system. Maize and soybean were selected as materials in the experiment, where every 2 rows of maize were intercropped with 2 rows of soybean.

**Results and discussion:**

The results showed that the combination of BC and N significantly affected the yield, WUE, NRE and quality of intercropped maize and soybean. The treatment of 15 t ha^-1^ BC and 180 kg ha^-1^ N increased grain yield and WUE, while that of 15 t ha^-1^ BC and 135 kg ha^-1^ N enhanced NRE in both years. Nitrogen promoted the protein and oil content of intercropped maize, but decreased the protein and oil content of intercropped soybean. BC did not enhance the protein and oil content of intercropped maize, especially in the first year, but increased maize starch content. BC was found to have no positive impact on soybean protein, but it unexpectedly increased soybean oil content. The TOPSIS method revealed that the comprehensive assessment value first increased and then declined with increasing BC and N application. BC improved the performance of maize-soybean intercropping system in terms of yield, WUE, NRE, and quality while N fertilizer input was reduced. The highest grain yield in two years was achieved for BC of 17.1-23.0 t ha^-1^ and N of 156-213 kg ha^-1^ in 2021, and 12.0-18.8 t ha^-1^ BC and 161-202 kg ha^-1^ N in 2022. These findings provide a comprehensive understanding of the growth of maize-soybean intercropping system and its potential to enhance the production in northeast China.

## 1 Introduction

Intercropping maize and soybean is considered as an effective method for improving land productivity in China (Zhang et al., 2008). This practice promotes farm biodiversity and resource efficiency, including land, nutrients, light and water (Ahmed et al., 2018; Feng et al., 2019; Iqbal et al., 2019; Raza et al., 2019a; Raza et al., 2019b). However, the spatial distributions of soil water and fertilizer vary greatly because of maize-soybean intercropping (Luan et al., 2021; Luo et al., 2022). Moreover, the nutrient supply of the system falls short of demand owing to such factors as interspecific competition in the middle and late crop growth stages (Raza et al., 2022), which may result in crop failure (Duchene et al., 2017). Because of this, it is generally considered for farmers that high fertilizer application improves crop growth and grain yield. But the fact is that the practice reduces the intercropped dominance, and negatively affects biological N fixation of intercropped legume (Zhou and Butterbach-Bahl, 2014), and also has an adverse effect on inter-specific competition between the intercropped crops. Therefore, it is important for the intercropping system to increase soil nutrient supply continuously during the critical period to balance resource competition and reach the aim of N fertilizer reduction and efficiency increase.

Biochar (BC) is a nutrient-rich solid and insoluble organic compound. It is produced by the thermal decomposition of organic matter at the temperature between 200 and 1200°C under the anaerobic condition (Weber and Quicker, 2018; Farhangi-Abriz et al., 2021). BC possesses high porosity, a large specific surface area, and a strong adsorption capacity (Alkharabsheh et al., 2021). Although it lacks effective N content, it plays a crucial role in influencing soil nitrogen’s effectiveness by directly or indirectly impacting such process as nitrification, mineralization, and N fixation (Jindo et al., 2020). When the practice of combined BC and compound fertilizer application is conducted, it can improve nutrient use efficiency through achieving slow-release nutrients from BC (Mohammad et al., 2022). Previous researches (Seleiman et al., 2020; Solaiman et al., 2020; Wang et al., 2020) also indicate that BC combined with N fertilizer raises soil fertility, promotes soil aggregation, enhances plant N uptake and ultimately improve yield, water use efficiency (WUE), and N recovery efficiency (NRE). However, the optimal amounts of BC and N fertilizer vary depending on the crop’s nutrient requirements and combination ratio. To maximize crop growth and yield, it is crucial to apply BC and N fertilizer at suitable rates based on crop nutrient requirements and soil properties. For example, the application of 5 t ha^-1^ of BC with 50 kg ha^-1^ of N fertilizer is recommended for achieving maximum N fertilizer use efficiency, while the application of 0 t ha^-1^ of BC and 100 kg ha^-1^ of N fertilizer is suggested to obtain the highest maize yield (Omara et al., 2020). Jin et al. (2019) found that 40 t ha^-1^ BC combined with 90 kg ha^-1^ N fertilizer resulted in the highest rapeseed yield, while 20 t ha^-1^ BC combined with 90 kg ha^-1^ N fertilizer to the utmost degree improved soil available phosphorus content in the rapeseed field. To improve efficiency, ensure crop yield, and save costs, it is essential to have scientific guidance on appropriate BC and N fertilizer applications (Sun et al., 2019). At present, mathematical models have been utilized to provide guidance for the application of water and fertilizer in agriculture (He et al., 2021a; He et al., 2021b), there has been limited research on mathematical models about BC and N fertilizer coupling. Additionally, current research on the combination of BC and N fertilizer mainly focuses on monoculture, with limited studies on intercropping systems, particularly regarding the comprehensive regulation and analysis of intercropping yield, water, and N utilization. Therefore, it is necessary to determine the optimum application of BC and N for maize-soybean intercropping systems. This will not only increase crop yield and enhance water and N utilization but also implement the synergistic effect of BC and N, leading to increased efficiency and cost savings.

The objective of this study is to address several important questions regarding the optimal use of BC and N fertilizer in maize-soybean intercropping systems in northeast China. Specifically, the study aims to investigate how various combinations of BC and N fertilizer impact crop yield, WUE, NRE, and quality. In addition, the study seeks to establish a systematic evaluation model for the intercropping system and determine the optimal application of BC and N for maximizing the benefits of the intercropping system.

## 2 Materials and methods

### 2.1 Experimental site

**Figure 1:**
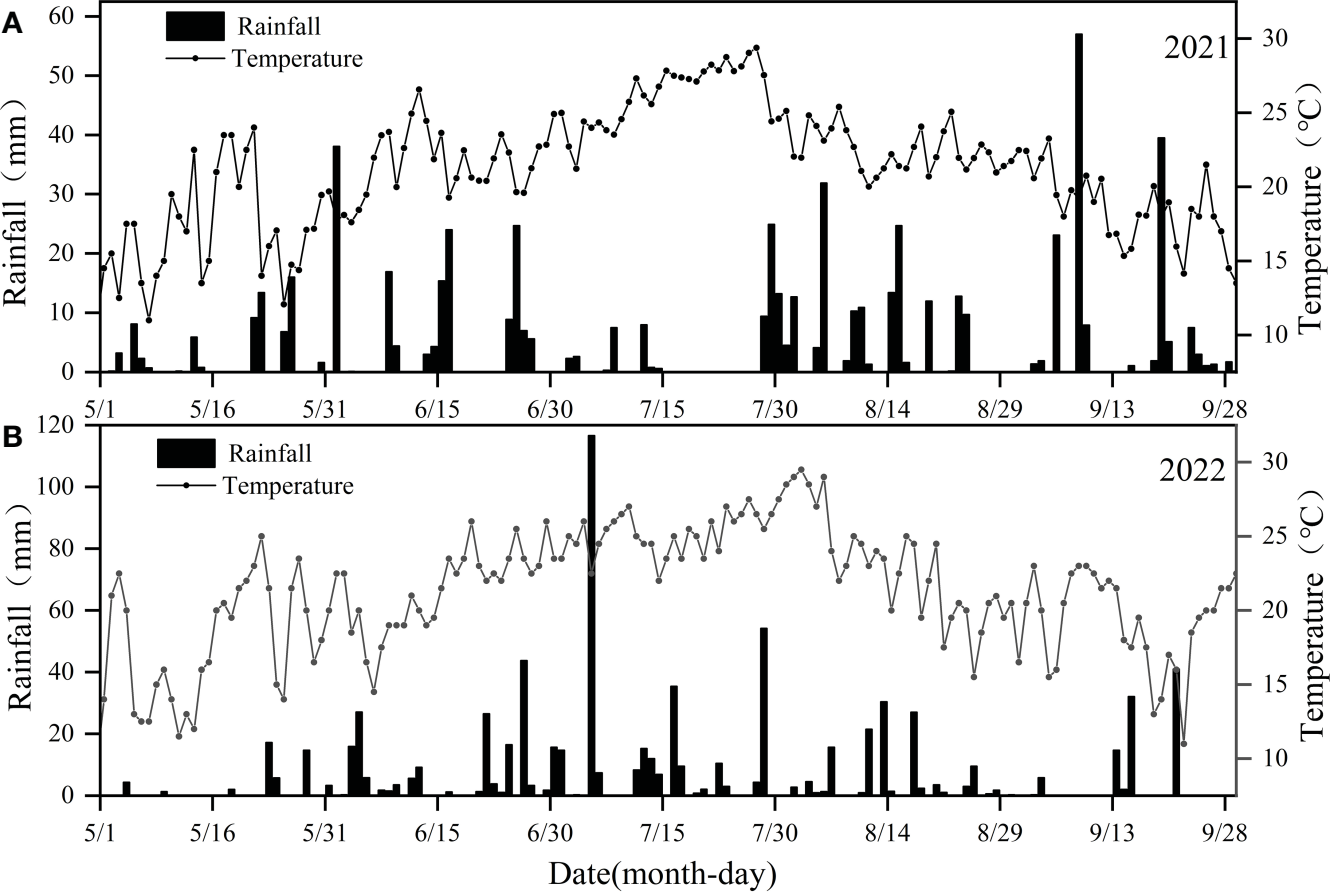
Rainfall and daily average temperature during the period of test. **(A)** stands for rainfall and daily average temperature during the period of test in 2021, **(B)** stands for rainfall and daily average temperature during the period of test in 2022.

Two-year field experiments in the experimental station of Water Conservancy College, Shenyang Agricultural University, China (41°84′N, 123°57′E, and altitude of 44.7 m) were carried out from May to September 2021 and 2022. The experimental site is characterized as temperate continental semi-humid monsoon climate type, with simultaneous precipitation and heat. There is about 78% of annual precipitation occurring from June to September in the site. **Figure 1** shows daily rainfall and daily average temperature during the growth period of intercropped crops, when total rainfall and average temperature were 596.2 mm and 9.0 °C in 2021 (**Figure 1A**), while they were 796.1 mm and 8.7 °C in 2022 (**Figure 1B**). The soil type of this site is brown soil, of which the physical and chemical properties are presented in **Table 1**.

**Table 1:** Basic physical and chemical properties of soil.

Total nitrogen (g·kg^-1^)	Total phosphorus (g·kg^-1^)	Total potassium (g·kg^-1^)	Soil organic matter (g·kg^-1^)	0~90cm volume weight of soil (g·cm^-3^)		Field water holding capacity (cm^3^·cm^-3^)	wilting coefficient (cm^3^·cm^-3^)
0.67	0.47	23.19	33.93	1.42	0.38		0.18

### 2.2 Experimental design

**Figure 2:**
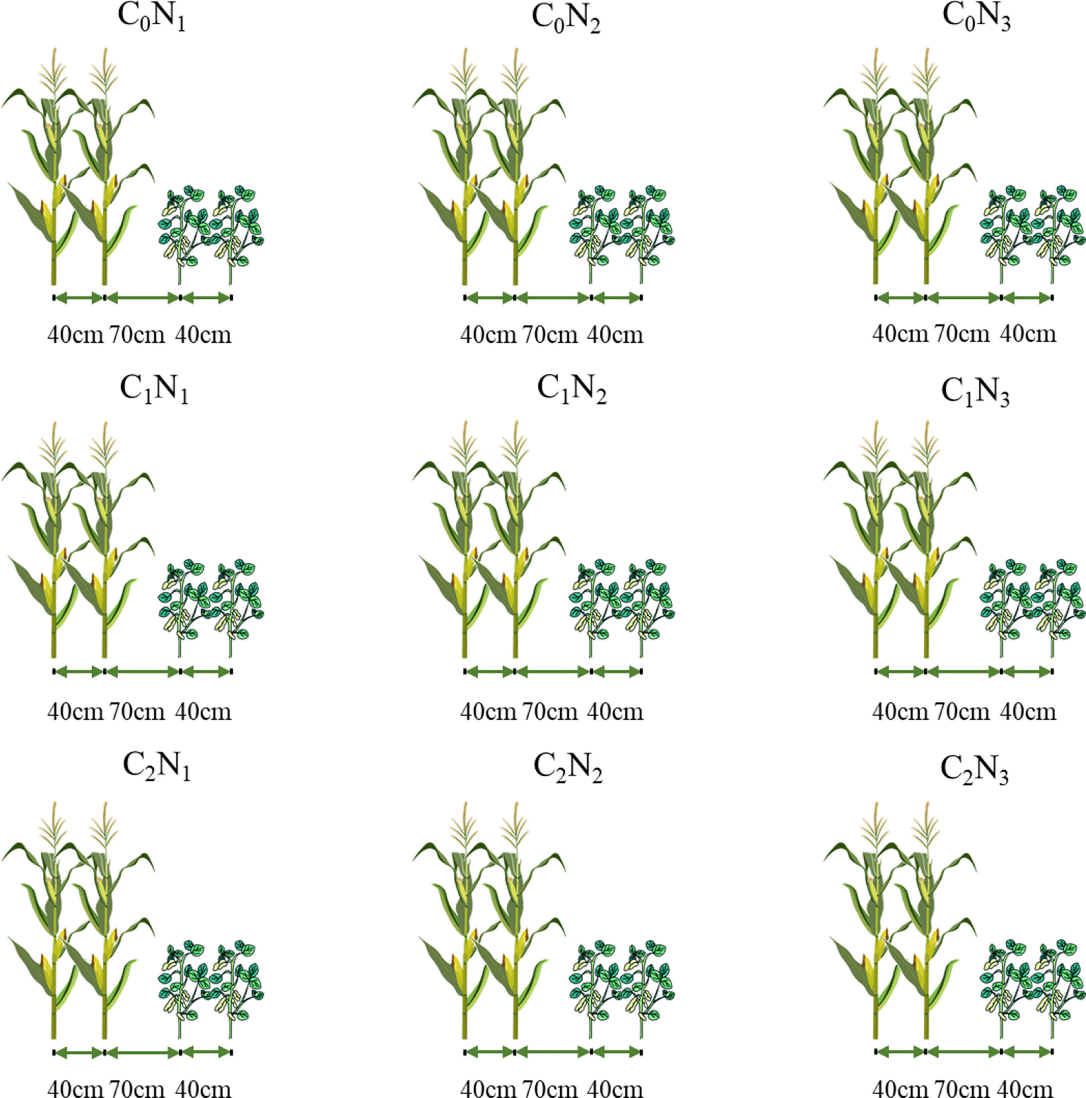
Schematic diagram of maize-soybean intercropping. BC application rates: 0 t ha^-1^ (C_0_), 15 t ha^-1^ (C_1_) and 30 t ha^-1^ (C_2_); N application rates: 135 kg ha^-1^ (N_1_), 180 kg ha^-1^ (N_2_), 225 kg ha^-1^ (N_3_). BC was mixed uniformly into the soil at a depth of 30 cm before sowing in 2021 but was not applied in the following year. N fertilizer was applied before sowing in the two years.

Maize (*Zea mays* L., cv. Zhengdan 958) and soybean (*Glycine max* (Linn.) Merr., cv. Liaodou 32) were selected as materials in the experiment, where every 2 rows of maize were intercropped with 2 rows of soybean. The commercial BC, produced by pyrolyzing maize straw under the oxygen-free condition at a temperature of 450 °C, was purchased from Liaoning Jinhefu Agricultural Development Company. Its basic physical and chemical properties are given in **Table 2**. BC was mixed uniformly into the soil at a depth of 30 cm before sowing in 2021 but was not applied in the following year. BC application rates were 0 t ha^-1^ (C_0_), 15 t ha^-1^ (C_1_) and 30 t ha^-1^ (C_2_), while N application rates were 135 kg ha^-1^ (N_1_), 180 kg ha^-1^ (N_2_), 225 kg ha^-1^ (N_3_). Among them, N_3_ is the conventional N application, which was used as control for N application, and the applications of 180 kg ha^-1^ and 135 kg ha^-1^ pointed at N reduction by 20% and 40% of N_3_. The experiment in 2021 was laid out in an orthogonal design L9 (3^2^) (**Figure 2**) by taking C_0_N_3_ as the control treatment with three replications, as was that in 2022.

**Table 2:** Basic indicators of BC.

Total nitrogen (g·kg^-1^)	Total phosphorus (g·kg^-1^)	Total potassium (g·kg^-1^)	organic biochar (g·kg^-1^)	pH
10.2	8.1	15.7	515.0	8.5

There were 27 experimental plots, each of which was 18 m^2^ (6 m × 3 m) in size. The distance between rows was 40 cm in maize or in soybean, and that between adjacent maize and soybean rows was 70 cm (**Figure 2**). The planting spacing in the maize row was 15 cm, while that in the soybean row was 20 cm. Besides, a 90 kg ha^-1^ of P_2_O_5_ and 120 kg ha^-1^ of K_2_O were applied evenly with N to guarantee adequate nutrition in the experimental field fertilizer before sowing in the two years.. All treatments were under the normal field management and only natural rainfall was available without any irrigation during the two growing periods. Maize and soybean were simultaneously sown on 6 May, 2021 and on 2 May, 2022, and were harvested on 25 September, 2021 and 27 September, 2022.

### 2.3 Measurements

#### 2.3.1 Grain yield and quality

The grain yield of all maize and soybean plants in each plot was manually harvested at physiological maturity. Subsequently, their grain quality, including protein, oil content and starch, was analyzed using the FOSS near-infrared grain quality analyzer (Infratec 1241, Foss company, Denmark). It must be noted that all quality indicators except for soybean starch could be measured by the instrument, Despite this, soybean protein and oil content could also to some degree reflect its quality.

#### 2.3.2 Water consumption and water use efficiency

Soil water content was measured to a depth of 100 cm at 10 cm increments by a soil auger using the oven-dried method before sowing and at harvest. A set of three probes was installed manually at a depth of 100 cm in the middle of the rows between maize and maize, maize and soybean, soybean and soybean in each plot.

Water consumption was calculated as follows:


(1) ET=I+P−ΔW−R−D+K

Where *ET* is water consumption; *W*_T_ is irrigation amount; *P* is effective rainfall in the growth stage; *ΔW* is the difference of water storage in the soil planned wetting layer between the beginning and end of the period; R is surface runoff; *D* is deep leakage; *K* is groundwater supply. W_T_ was zero because there was no irrigation in the experiment. Additionally, the experimental plot was flat, and hence there was no surface runoff loss, namely *R*=0. Soil moisture content at a depth of 90 cm to 100 cm did not vary significantly, which represented no deep leakage (*D*=0). Deeper groundwater depth was available, which means that there was no groundwater supply (K=0).

*Δ*W was calculated as follows:


(2) $$\Delta W=\frac{\theta_{m}\rho_{b}h}{\rho_{w}}$$

Where *θ*_m_ is 0-100 cm soil mass moisture content (%); h is soil thickness (cm); *ρ*_b_ is average soil bulk density at the depth of 0 ~ 100 cm soil layer (g/cm^3^), *ρ*_w_ is water density (g/cm^3^).

WUE was calculated as follows:


(3) $$WUE=\frac{\text{Y}}{\text{ET}}$$

Where *Y* is crop yield (kg ha^-1^).

#### 2.3.3 Nitrogen fertilizer recovery efficiency

N concentration of straw and grain was determined using Auto Kjeldahl Analysis Equipment (KJELTEC 2300, Foss company, Denmark) according to the Kjeldahl method. The aboveground N uptake was equal to straw N uptake and grain N uptake. NRE was defined as:


(4) $$\text{NRE=}\frac{\text{Above ground N uptake}\left(\text{fertilizer}\right)\text{-Above ground N uptake(unfertilized)}}{\text{Total fertilizer N applied}}\times 100$$

### 2.4 Comprehensive evaluation system framework

Two steps were involved to conduct the comprehensive evaluation. One was to categorize the factors and then establish a hierarchical structure of a system. Another was to analyze the relationships among the factors in the system in order to gain a better understanding of the system as a whole.

#### 2.4.1 Evaluation factor set and its sub-factor set construction

(1) All indices of maize and soybean were divided into yield index (*u*_1_), efficiency index (*u*_2_) and quality index (*u*_3_).


(5) $$U_{i}=\{u_{1},u_{2},u_{3}\}$$

(2) All secondary indices were categorized and determined as sub-factors. Maize yield (*u*_11_) and soybean yield (*u*_12_) were categorized as yield indicators, WUE (*u*_21_) and NRE (*u*_22_) were categorized as efficiency indicators, Maize protein (*u*_31_), maize starch (*u*_32_), maize oil (*u*_33_), soybean protein (*u*_34_), soybean oil (*u*_35_) were categorized as quality indicators.


(6) $$u_{ij}\to \left(\begin{array}{c} u_{1}=\left\{u_{11},u_{12}\right\}\\ \begin{array}{c} u_{2}=\left\{u_{21},u_{22}\right\}\\ u_{3}=\left\{u_{31},u_{32},u_{33},u_{34},u_{35}\right\} \end{array} \end{array}\right)$$

**Figure 3:**
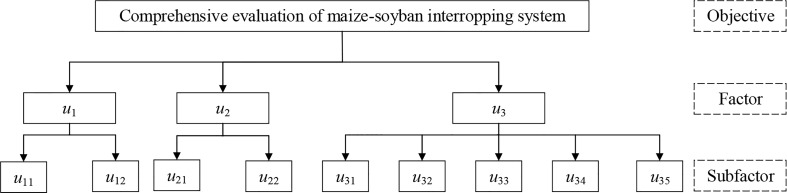
Maize-soybean intercropping system comprehensive evaluation hierarchical model.

The specific hierarchical model is shown in **Figure 3**.

#### 2.4.2 Comprehensive evaluation of TOPSIS

##### 2.4.2.1 Factor weight determination

An evaluation system is constructed to determine the subjective weight on basis of analytic hierarchy process (AHP). And the questionnaire is used to score each index (1-9 points) in pairs to compare the importance of the factors. Consistency ratio (C_R_) is then used to check the acceptability of the matrix. When C_R_ is less than 0.10, the consistency test is considered to pass, and the judgment matrix is acceptable. The specific calculating procedure is in light of the literatures of Sahoo et al. (2018) and Kundu et al. (2017).

##### 2.4.2.2 Sub factor weight determination

The entropy method is selected to calculate the objective weight. It is able to effectively reflect the information implied by the data and exhibit strong operability (Zhong et al., 2017). The detailed calculating procedure is according to the literatures of Wang et al. (2015); Hou et al. (2018) and Liu et al. (2013).

##### 2.4.2.3 Combination weight determination

The combined weighting evaluation method is selected in order to better balance the subjective and objective demand and improve the reliability and scientific property of weight distribution based on the game theory. It is used to determine the comprehensive weight of a single index of maize-soybean intercropping, that is, a weight set is constructed based on the two weights of the subjective AHP and objective entropy,:u_k_={u_k1_,u_k2_, …, u_kn_}(k=1,2).


(7) $$\text{u}=\sum_{k=1}^{L}\alpha_{\text{k}}{\text{uk}}_{\;}^{\text{ T}}(\alpha_{\text{k}}>0,\sum_{k=1}^{L}\alpha_{\text{k}}=1)$$

Where u is weight vector of weight set; α_k_ is linear combination coefficient.

The game theory method is used to get the combination weight.


(8) $${\text{Wj}}_{\;}^{\;*}=\sum_{\text{j}=1}^{\text{n}}{\text{w}}_{1j}{\text{w}}_{2\text{j}}^{\text{ T}},\text{j}=1,2,\ldots ,\text{n}$$

Where w_1j_ is subjective weight determined based on AHP; w_2j_ is objective weight determined based on entropy weight method

##### 2.4.2.4 Euclidean distance determination

The original data matrix of m evaluation indicators and an evaluation objects is as follows:


(9) $$\left[\begin{array}{cc} \begin{array}{cc} x_{11} & x_{12}\\ x_{21} & x_{22} \end{array} & \begin{array}{cc} \ldots & x_{1n}\\ \cdots & x_{2n} \end{array}\\ \begin{array}{cc} \vdots & \vdots \\ x_{m1} & x_{m2} \end{array} & \begin{array}{cc} \ddots & \vdots \\ \ldots & x_{mn} \end{array} \end{array}\right]$$

The matrix is normalized as follows


(10) $$\text{Z}={\left[z_{ij}\right]}_{\text{m}\times \text{n}}$$

where


(11) $$z_{ij}=x_{ij}/\sqrt{\sum_{i=1}^{n}x_{ij}^{2}}$$

The maximum value Z^+^ is defined as ${\text{z}}_{1}^{\;+},{\text{z}}_{2}^{\;+},\cdot \cdot \cdot ,{\text{zm}}_{\;}^{\;+}$ and the minimum value Z^-^ is defined as ${\text{z}}_{1}^{\;-},{\text{z}}_{2}^{\;-},\cdot \cdot \cdot ,{\text{zm}}_{\;}^{\;-}$ .

The weighted distance is calculated between the *ith* (*i*=1, 2,···, n) evaluation object and positive ideal solution ${\text{Zj}}_{\;}^{\;+}$ or negative ideal solution ${\text{Zj}}_{\;}^{\;-}$ as follows:


(12) $$\;D_{i}^{+}=\sqrt{\sum_{j=1}^{m}w_{j}^{*}{\left(Z_{j}^{+}-z_{ij}\right)}^{2}}\;$$

(13) $$\;D_{i}^{-}=\sqrt{\sum_{j=1}^{m}w_{j}^{*}{\left(Z_{j}^{-}-z_{ij}\right)}^{2}}$$

$D_{\text{i}}^{\;+}$ is positive ideal solution distance;. $D_{\text{i}}^{\;-}$ is negative ideal solution distance

The score *S_i_* of the *i*th evaluation object is calculated as follows:


(14) $$S_{i}=\frac{D_{i}^{-}}{D_{i}^{-}+D_{i}^{+}}$$

*S*_i_ is comprehensive evaluation score.

### 2.5 Data analysis

Microsoft Excel 2021 software was used to sort out data, and the plotting was performed by OriginPro 2023 software. All statistical analysis was conducted using SPSS (IBM, Chicago, USA). MATLAB 2021b software (MathWorks, MA, USA) was used to execute comprehensive evaluation.

## 3 Results

### 3.1 Yield

**Figure 4:**
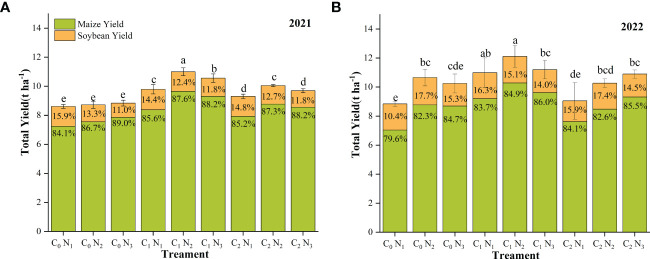
Yield of maize-soybean intercropping system in 2021 and 2022. **(A)** shows the yield in 2021 and **(B)** shows the yield in 2022. Different lowercase letters indicate significant differences among different treatments (P<0.05). The column bars and error bars represent the mean yield of three replicates and the standard deviation of the mean, respectively. BC application rates: 0 t ha^-1^ (C_0_), 15 t ha^-1^ (C_1_) and 30 t ha^-1^ (C_2_); N application rates: 135 kg ha^-1^ (N_1_), 180 kg ha^-1^ (N_2_), 225 kg ha^-1^ (N_3_). BC was mixed uniformly into the soil at a depth of 30 cm before sowing in 2021 but was not applied in the following year. N fertilizer was applied before sowing in the two years.

BC and N application significantly affected the yield of maize-soybean intercropping system in 2021 (**Figure 4A**) and 2022 (**Figure 4B**). The C_2_ and C_1_ treatments increased maize yield by 8.76%–23.3% and 3.23%–30.8%, respectively, compared with C_0_. The soybean yield showed a declining trend with an increase in N addition in 2021, while in 2022, while soybean yield increased at first and then decreased with increasing application rate. Additionally, soybean yield in 2022 was higher than soybean yield in 2021. The highest total yield was for C_1_N_2_, 11.0 t ha^-1^ in 2021 and 12.1 t ha^-1^ in 2022. The application of BC increased the yield of the maize-soybean intercropping system, but the yield increase effect of C_2_ was not as good as that of C_1_.

### 3.2 Water use efficiency

BC and N application had a significant impact on ET, but their interaction had no significant effect on it (**Table 3**). The effect of BC in 2021 was slightly smaller than that of N fertilizer, on the contrary, its effect is much greater than that of N application in the next year. With increase in N application, ET showed an increasing trend, while it decreased at first and then increased as BC addition was raised. The maximum ET was noticed at C_0_N_3_ and the minimum one was for C_1_N_1_. It indicated that intercropped crops consumed more soil water under the condition of the conventional N addition. BC addition combined with reduced N decreased ET but BC was always negatively correlated with ET. BC and N application also had a significant impact on WUE, but the interaction between BC and N was not significant in the second year. WUE of C_1_N_2_ was significantly higher than that of other treatments. Thus, BC combined with reduced N fertilization led to reduction in ET and increase in WUE of maize-soybean intercropping system, but high BC addition could not keep the increasing trend of WUE.

**Table 3:** ET and WUE under different combinations of BC and N levels.

Treatment	ET(mm)		WUE(kg·ha^-1^·mm^-1^)	
	2021	2022	2021	2022
C_0_N_1_	664.37 ± 5.49c	830.32 ± 1.98ab	12.98 ± 0.27e	10.65 ± 0.27c
C_0_N_2_	670.42 ± 2.63ab	832.41 ± 2 08a	13.00 ± 0.40e	12.81 ± 0.40b
C_0_N_3_	672.73 ± 3.41a	834.65 ± 2.21a	13.13 ± 0.32e	12.28 ± 0.32b
C_1_N_1_	657.99 ± 2.22abc	812.24 ± 1.68e	14.88 ± 0.54c	13.53 ± 0.54ab
C_1_N_2_	664.37 ± 2.92bc	813.05 ± 3.43de	16.57 ± 0.38a	14.90 ± 0.38a
C_1_N_3_	665.68 ± 4.00abc	818.12 ± 3.17cde	15.86 ± 0.40b	13.68 ± 0.40ab
C_2_N_1_	662.68 ± 4.09bc	817.28 ± 2.82cde	14.05 ± 0.30d	11.09 ± 0.30c
C_2_N_2_	665.17 ± 4.26a	821.15 ± 5.58cd	15.12 ± 0.17c	12.51 ± 0.17b
C_2_N_3_	667.46 ± 6.41bc	822.25 ± 4.87bc	14.52 ± 0.88cd	13.26 ± 0.88b
C	3.69^*^	46.32^*^	144.71^**^	24.06^**^
N	4.37^*^	3.52^*^	16.64^**^	14.25^**^
C·N	0.16	0.22	4.70^**^	2.39

Values ​​represent mean ± standard deviation of 3 replicates. ^*^ Indicates that the effect is significant at a level less than 0.05, and ^**^ indicates that it is extremely significant at a level less than 0.01. BC application rates: 0 t ha^-1^ (C_0_), 15 t ha^-1^ (C_1_) and 30 t ha^-1^ (C_2_); N application rates: 135 kg ha^-1^ (N_1_), 180 kg ha^-1^ (N_2_), 225 kg ha^-1^ (N_3_). BC was mixed uniformly into the soil at a depth of 30 cm before sowing in 2021 but was not applied in the following year. N fertilizer was applied before sowing in the two years.

### 3.3 Nitrogen uptake and nitrogen recovery efficiency

BC and N application had a significant impact on straw and grain N absorption, but the effect of BC application was greater than that of N application (**Table 4**). Maize straw and grain N uptake showed an overall increasing trend with increase in N application. However, total N uptake increased at first and then decreased with increasing BC application. Total N uptake of the C_1_ treatments increased by approximately 4.03%~31.8% in 2021 and about 11.1%~19.5% in 2022 compared with that of the C_0_ treatments. Among the treatments with the same BC level, the highest grain N uptake was observed for C_1_ treatments while C_2_ treatments had the maximum straw N uptake, indicating that excessive BC application was unhelpful for N transporting from straw to grain. NRE declined with increase in N application under C_0_ and C_1_ treatments, but this trend was not observed for C_2_ treatments. Therefore, reduction in N fertilizer input was an effective way to improve NRE, but excessive BC application probably weakened the effect.

**Table 4:** N uptake and NRE under different combinations of BC and N levels.

Treatment	Straw N uptake (kg ha^-1^)		grain N uptake (kg ha^-1^)		total N uptake (kg ha^-1^)		NRE (%)	
	2021	2022	2021	2022	2021	2022	2021	2022
C_0_N_1_	55.87 ± 3.06d	56.41 ± 0.76cd	54.97 ± 1.50d	58.57 ± 3.78c	110.83 ± 2.56e	114.99 ± 4.54d	33.25 ± 0.57cd	34.40 ± 2.88bc
C_0_N_2_	53.98 ± 3.73d	58.75 ± 1.96cd	56.52 ± 0.88d	58.92 ± 2.51c	110.50 ± 3.51e	117.34 ± 4.14d	26.23 ± 1.95e	27.31 ± 2.103cd
C_0_N_3_	60.91 ± 4.05bcd	53.95 ± 1.38d	67.01 ± 3.94bc	68.42 ± 2.11b	127.92 ± 2.94d	122.37 ± 1.86d	28.73 ± 1.31de	23.67 ± 2.33cd
C_1_N_1_	67.76 ± 2.20ab	62.66 ± 1.68bcd	71.57 ± 3.28b	69.87 ± 1.52b	139.33 ± 5.47bc	132.53 ± 2.51c	50.68 ± 3.23a	47.65 ± 6.16a
C_1_N_2_	62.54 ± 1.94bc	58.19 ± 1.65cd	83.07 ± 2.27a	84.83 ± 4.26a	145.60 ± 4.11ab	143.03 ± 3.72b	45.74 ± 2.28a	41.45 ± 5.52ab
C_1_N_3_	71.38 ± 1.07a	60.98 ± 5.75bcd	80.60 ± 2.27a	84.39 ± 2.75a	151.98 ± 5.39a	145.37 ± 2.06ab	39.42 ± 2.40b	34.07 ± 2.59bc
C_2_N_1_	59.63 ± 3.85d	65.99 ± 1.91bc	55.66 ± 3.93d	56.46 ± 1.81c	115.30 ± 5.58e	122.44 ± 3.61d	38.53 ± 4.14bc	39.73 ± 4.49ab
C_2_N_2_	73.35 ± 2.50a	69.69 ± 2.65ab	61.30 ± 3.43cd	61.64 ± 2.78c	134.65 ± 4.20cd	131.33 ± 3.42c	39.65 ± 2.34b	34.81 ± 2.34bc
C_2_N_3_	74.97 ± 3.88a	80.13 ± 5.05a	64.21 ± 4.16c	71.27 ± 3.35b	139.18 ± 6.78bc	151.40 ± 6.25a	33.73 ± 3.56cd	36.82 ± 3.45b
C	27.45^**^	21.12^**^	78.53^**^	116.28^**^	80.16^**^	73.04^**^	81.80^**^	22.33^**^
N	10.64^**^	1.05	17.20^**^	50.06^**^	29.94^**^	37.08^**^	15.29^**^	11.57^**^
C·N	5.44^**^	2.99^*^	2.73^*^	6.51^**^	3.77^*^	7.19^**^	4.13^**^	1.63

Values ​​represent mean ± standard deviation of 3 replicates. * Indicates that the effect is significant at a level less than 0.05, and ** indicates that it is extremely significant at a level less than 0.01. BC application rates: 0 t ha^-1^ (C_0_), 15 t ha^-1^ (C_1_) and 30 t ha^-1^ (C_2_); N application rates: 135 kg ha^-1^ (N_1_), 180 kg ha^-1^ (N_2_), 225 kg ha^-1^ (N_3_). BC was mixed uniformly into the soil at a depth of 30 cm before sowing in 2021 but was not applied in the following year. N fertilizer was applied before sowing in the two years.

### 3.4 Maize and soybean quality

**Figure 5:**
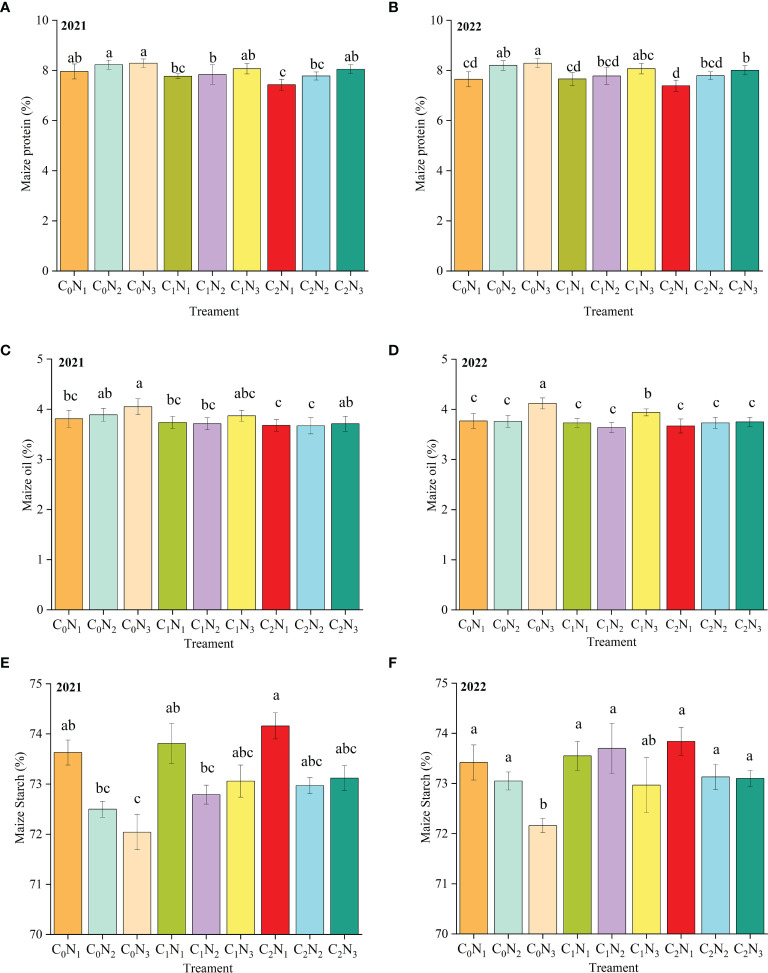
Maize quality in 2021 and 2022. **(A, B)** show maize protein content in 2021 and 2022, respectively. **(C, D)** show maize oil content in 2021 and 2022 respectively, and **(E, F)** show maize starch content in 2021 and 2022 respectively. Different lowercase letters in the same column mean significant differences among different treatments at 0.05 level. The column bars and error bars represent the mean maize quality of three replicates and the standard deviation of the mean, respectively. BC application rates: 0 t ha^-1^ (C_0_), 15 t ha^-1^ (C_1_) and 30 t ha^-1^ (C_2_); N application rates: 135 kg ha^-1^ (N_1_), 180 kg ha^-1^ (N_2_), 225 kg ha^-1^ (N_3_). BC was mixed uniformly into the soil at a depth of 30 cm before sowing in 2021 but was not applied in the following year. N fertilizer was applied before sowing in the two years.

**Figure 6:**
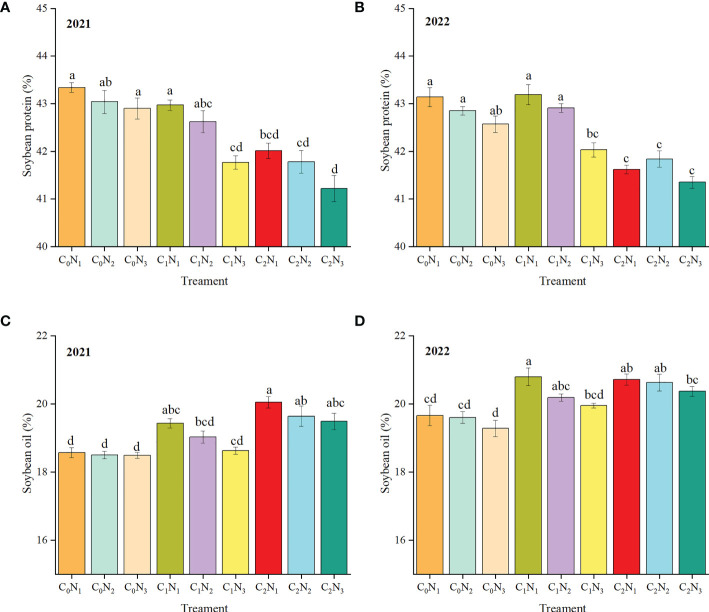
Soybean quality in 2021 and 2022. **(A, B)** show soybean protein content in 2021 and 2022, respectively. **(C, D)** show soybean oil content in 2021 and 2022, respectively. Different lowercase letters in the same column mean significant differences among different treatments at 0.05 level. The column bars and error bars represent the mean soybean quality of three replicates and the standard deviation of the mean, respectively. BC application rates: 0 t ha^-1^ (C_0_), 15 t ha^-1^ (C_1_) and 30 t ha^-1^ (C_2_); N application rates: 135 kg ha^-1^ (N_1_), 180 kg ha^-1^ (N_2_), 225 kg ha^-1^ (N_3_). BC was mixed uniformly into the soil at a depth of 30 cm before sowing in 2021 but was not applied in the following year. N fertilizer was applied before sowing in the two years.

The impact of BC and N application on the quality of intercropped maize and soybean is presented in **Figures 5** and **6**. Maize protein (**Figures 5A, B**) and maize oil content (**Figures 5C, D**) decreased with an increase in BC application, although the variations in the two years were slightly different. The first year of BC application did not have a positive effect on protein and oil content of maize grain. However, BC had little effect on oil content in second year while protein content continued to decline. C_0_N_3_ resulted in the highest protein and oil content of maize. And maize starch content (**Figures 5E, F**) increased with the rising BC application. BC was found to have no positive impact on soybean protein (**Figures 6A, B**) but it unexpectedly increased soybean oil content (**Figures 6C, D**).

### 3.5 Comprehensive growth evaluation

The weight of the model is determined by AHP method (**Supplementary Table 1**) and entropy weight method (**Supplementary Table 2**). respectively. The obtained two sets of weights are combined through the principle of game theory (**Supplementary Table 3**). The combined game theory weight was then utilized to calculate the ideal solution and comprehensive evaluation score by the TOPSIS comprehensive model (**Table 5**). The highest overall evaluation score was for C_1_N_2_, followed by C_1_N_3_ in 2021 and C_1_N_1_ in 2022. This meant that slight N fertilizer input in the second year was more beneficial for the overall growth of the intercropping system under the condition of C_1_. The lowest scores were different over the two years, C_0_N_2_ in 2021 and C_0_N_1_ in 2022.

**Table 5:** Comprehensive growth evaluation based on TOPSIS.

Treatments	D^+^		D^-^		S		Rank	
	2021	2022	2021	2022	2021	2022	2021	2022
C_0_N_1_	0.498	0.446	0.232	0.231	0.318	0.341	8	9
C_0_N_2_	0.506	0.319	0.176	0.309	0.258	0.492	9	5
C_0_N_3_	0.490	0.366	0.235	0.305	0.324	0.454	7	7
C_1_N_1_	0.277	0.221	0.393	0.409	0.586	0.649	3	2
C_1_N_2_	0.234	0.223	0.489	0.472	0.676	0.679	1	1
C_1_N_3_	0.254	0.241	0.410	0.358	0.617	0.597	2	3
C_2_N_1_	0.381	0.440	0.296	0.235	0.437	0.348	6	8
C_2_N_2_	0.293	0.313	0.366	0.282	0.555	0.473	4	6
C_2_N_3_	0.341	0.279	0.297	0.324	0.465	0.537	5	4

BC application rates: 0 t ha^-1^ (C_0_), 15 t ha^-1^ (C_1_) and 30 t ha^-1^ (C_2_); N application rates: 135 kg ha^-1^ (N_1_), 180 kg ha^-1^ (N_2_), 225 kg ha^-1^ (N_3_). $D_{\text{i}}^{\;+}$ is the positive ideal solution distance; $D_{\text{i}}^{\;-}$ is the negative ideal solution distance; S_i_ is the comprehensive evaluation score.

### 3.6 Mathematical model construction

The comprehensive evaluation scores obtained above were used to conduct the quadratic polynomial stepwise regression, and then got the regression equation between BC-N application amount and comprehensive evaluation indicator.


(15) $$y_{1}=0.6600-0.2372x_{1}^{\;2}+0.0922x_{1}-0.0473x_{2}^{\;2}+0.0085x_{2}+0.0045x_{1}x_{2}$$

(16) $$y_{2}=0.6887-0.872x_{1}^{\;2}+0.0393x_{1}-0.2042x_{2}^{\;2}+0.0107x_{2}+0.0173x_{1}x_{2}$$

Where *x*_1_ and *x*_2_ reflect the coding values of N and BC application BC; *y*_1_ and *y*_2_ are the comprehensive assessment scores for 2021 and 2022.

The combined BC and N had a highly significant impact on the comprehensive assessment scores with a coefficient of determination (R^2^) of 0.944 for *y*_1_ and 0.933 for *y*_2_. Therefore, the regression model could be used to assess the impact of BC and N application on the overall growth of maize-soybean intercropping system. The positive and negative signs in front of *x*_1_ and *x*_2_ indicate promotion or inhibition, and the level of the coefficient indicates the strength of the influence. Both regression equations demonstrated that there was a positive role for BC or N application to promote the complete evaluation score, and the effect of BC was better. Additionally, the coefficient of *x*_1_ *x*_2_ is positive. That is to say BC and N had a positive coupling effect and both improved the comprehensive evaluation score.

**Figure 7:**
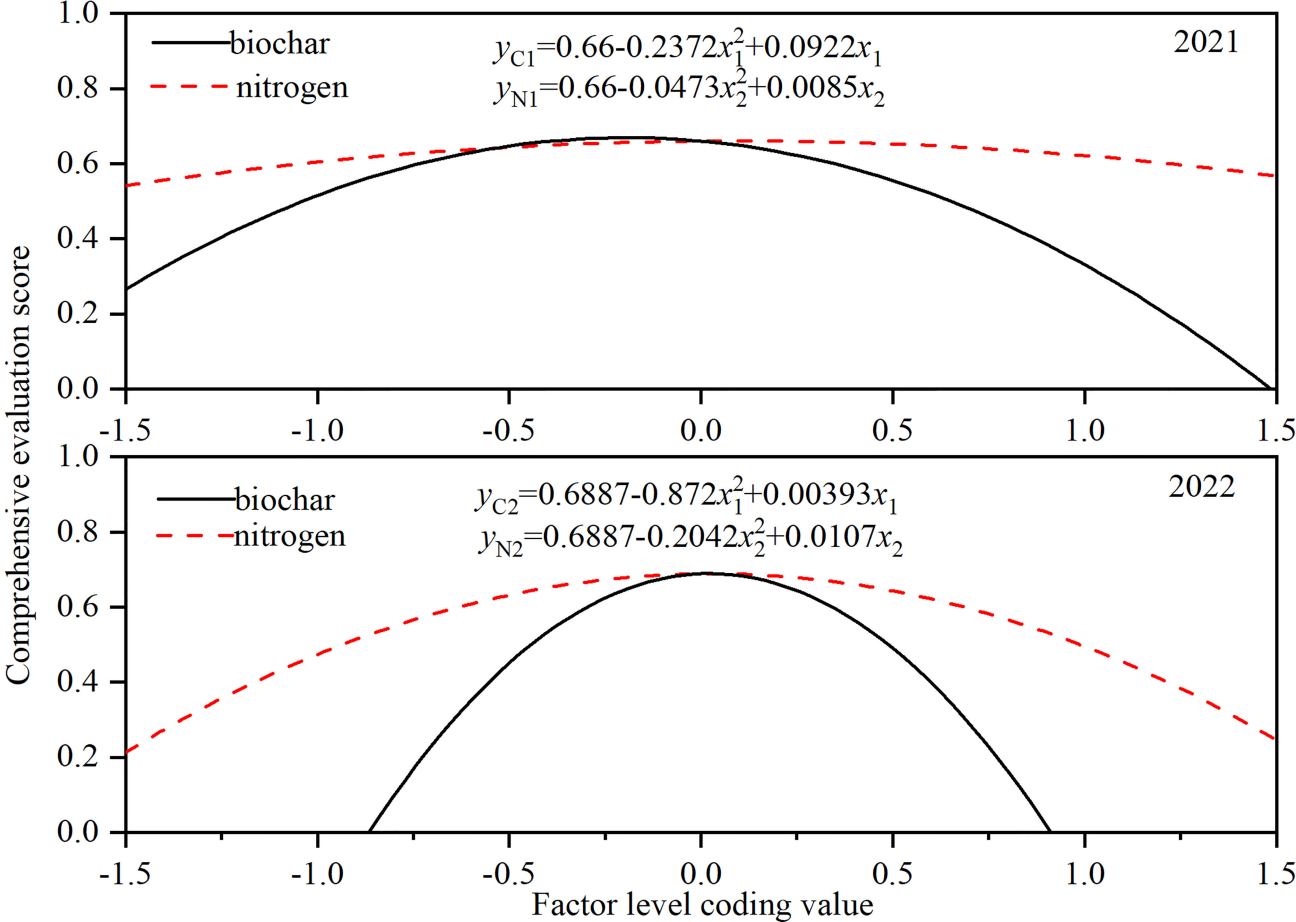
Effects of BC or N application on comprehensive evaluation score of maize-soybean intercropping system. y_C1_ and y_N1_ is the function of the single-factor effect function of on the comprehensive evaluation score in 2021, and y_C2_ and y_N2_ is single-factor effect function of comprehensive evaluation score in for 2022.

The reduced dimension was conducted to explore the impact of single factor-BC or N on the comprehensive evaluation score. **Figure 7** showed the comprehensive assessment value firstly increased and subsequently decreased as BC or N increased. The comprehensive evaluation score was maximum when BC coding value was 0.19 and N coding value was 0.09 in 2021. In 2022, the maximum comprehensive evaluation score was achieved when BC was 0.02 and N was 0.03N. Besides, the comprehensive growth assessment score for 2021 varied slowly with rising BC and N compared to that for 2022, which indicated that the intercropping growth was more sensitive to the application of BC and N in 2022.

**Figure 8:**
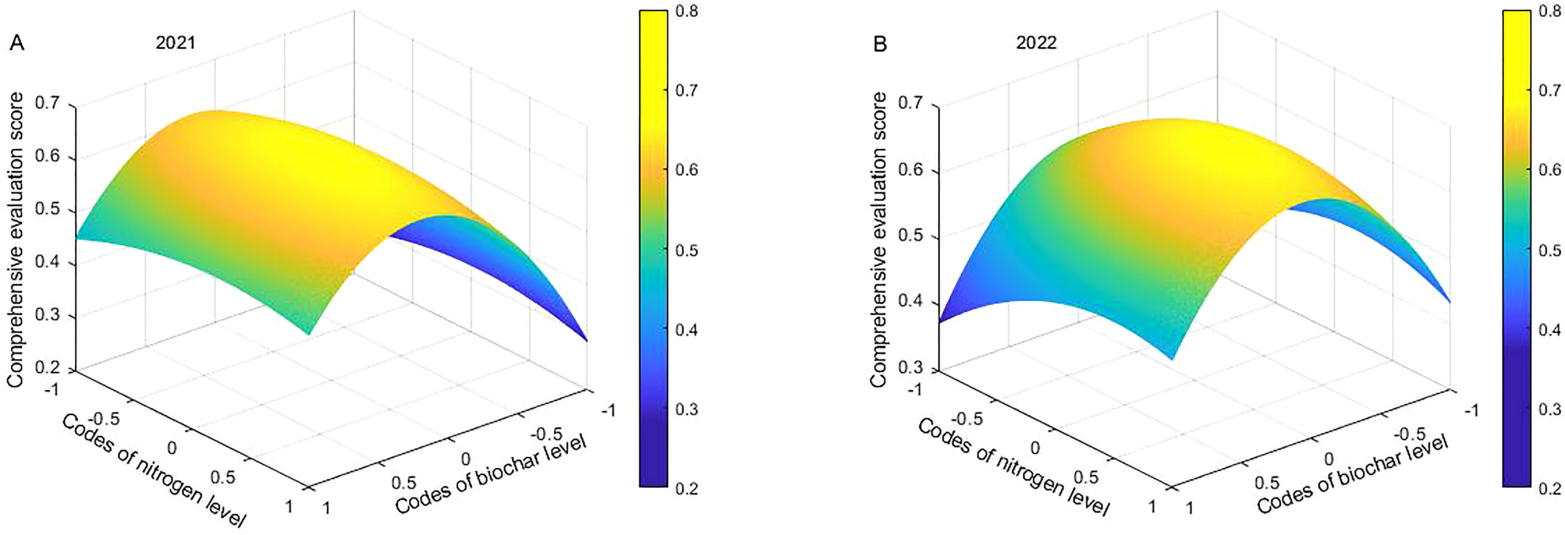
Coupling effect of BC and N on comprehensive evaluation score of Maize-Soybean intercropping system in 2021 and 2022.

The quadratic polynomials (Formula 15 and 16) were simulated for the optimum using Matlab2020b software (**Figure 8**). The optimal application range was determined by 90% of the maximum comprehensive score with the consideration of BC cost. The ranges were 0.14~0.53 for BC and -0.53~0.71 for N in 2021, and -0.20~0.25 for BC and -0.43~0.49for N in 2022. This is to say, the ranges of BC were 17.1~22.95 t ha^-1^ in 2021 and 12~18.75 t ha^-1^ in 2022, and those of N were 156.15~211.95 kg·ha^-1^ in 2021 and 160.65~202.05 kg·ha^-1^ in 2022.Therefore, the optimum combination was BC of 17.10~22.95 t ha^-1^ and N of 156.15~211.95 kg ha^-1^ in 2021, and 12~18.75 t ha^-1^ and 160.65~202.05 kg ha^-1^ in 2022.

## 4 Discussion

### 4.1 Combining BC with N fertilization increases crop yield

In this study, the combined application of BC and N resulted in increased yields in the maize-soybean intercropping system, to varying degrees compared to N application alone. This can be attributed to the ability of BC to absorb soil nutrients and release them slowly for crop absorption over an extended period (Purakayastha et al., 2019). In other words, N fertilizer supplements the nutrient deficiency of BC, while BC extends the retention time of fertilizer in the soil (Chen et al., 2018). However, the yield increase was higher in intercropping systems with lower BC compared to those with higher BC, as excessive BC increases soil C/N ratio, inhibiting soil microbial decomposition and N mineralization rate (Xia et al., 2022).

Intercropping, a traditional agricultural practice, offers several benefits including crop yield increase, resource utilization enhancement, pest and disease reduction, soil health improvement, and biodiversity increase (Mugi-Ngenga et al., 2022). However, intercropping systems also face challenges such as the negative impact of shade for intercropped soybean (Wu et al., 2016). Limited N fertilizer can restrict the growth of intercropped maize, thereby reducing the shading effect and significantly increasing soybean yield (Raza et al., 2019c). Moreover, reducing N fertilizer appropriately can increase maize’s absorption and utilization of soil N, enhance soybean nodulation and N fixation ability (Li et al., 2016), and ensure that both maize and soybean have sufficient N for growth with less N application (Xu et al., 2020). This study highlights the significant interaction between BC and N, indicating that incorporating BC into the soil can increase crop yield in situations where N fertilizer is limited. The synergistic or complementary interaction between BC and N improves soil nutrient availability and enhances slow-release performance of fertilizer (Chen et al., 2018). These findings are consistent with previous studies conducted by Guo et al. (2021) and Omara et al. (2020), suggesting that combined appropriate BC application and reduced N fertilizer can enhance the productivity of maize-soybean intercropping system.

### 4.2 Combining BC with N fertilizer improves ET and WUE

Our results indicated that BC combined with N fertilizer improved ET and WUE. The improvement of ET may be due to that small BC particles fill larger soil pores and hence alter soil water flux, which further decreases soil water depletion caused by both BC and N fertilizer application (Faloye et al., 2019). The significant effect of combined BC and N fertilizer addition on WUE improvement may be attributed to the impact of BC on ET, soil hydro-physical and chemical properties (Ajayi and Horn, 2016; Faloye et al., 2017). BC application enhances the relative water content of crop leaves, which leads to increased photosynthetic activity (Akhtar et al., 2014; Kammann et al., 2011). It also promotes water consumption in the early and mid-term grain filling, which is possibly related to stronger water absorption capacity of roots (Chen et al., 2010). The increase in soil moisture content in the plow layer and soil moisture holding capacity induced by BC addition, is a main mechanism for increasing crop yield (Jeffery et al., 2011; Rogovska et al., 2014). However, our results showed that excessive BC reduced the amplification of WUE. High BC level increases soil porosity and excess hydrophobic compound, resulting in soil water loss (Jeffery et al., 2015). Castellini et al. (2015) found that BC application from 15 to 30 t ha^-1^ reduced soil water holding capacity by 23.5% and increased water consumption, thereby decreasing WUE, which is consistent with our findings. Additionally, high BC darkens soil color and then increases its surface temperature, which reduces water viscosity and surface tension, accelerates soil water evaporation through radiation absorption (Yang et al., 2020; Wu et al., 2022). Therefore, only an appropriate combination of BC and N fertilizer can improve WUE of th intercropping systems.

### 4.3 Combining BC with N fertilizer promotes N uptake and improve NRE

Our results found that BC-N interaction increased crop N absorption, consistent with the findings of Ibrahim et al. (2020) who reported that combined BC and N acted as a sustained-release N fertilizer, providing sufficient N for crop growth. On the one hand, BC possesses a highly porous cellular structure and large specific surface area, enabling it to rapidly absorb N and other nutrients (Yao et al., 2017). This leads to the reduction in N leaching loss and improvement in mineral N, thereby increasing soil available N. BC application improves soil aeration by altering soil bulk density and porosity, and increases soil available water, which is beneficial for the alteration of soil N cycle and hence leads to greater soil mineralization and inorganic N availability (Xiao et al., 2016a; Xiao et al., 2016b). On the other hand, it is also helpful to promote maize roots growth and further improve N uptake through these roots. Additionally, higher N accumulation during the post-silking period under the BC-added conditions could not only reduce N remobilization from leaf to grain, but also maintain functional stay-green leaf, and thus promote dry matter accumulation and enhance grain yield (Lee and Tollenaar, 2007).

BC promotes plant N accumulation before and after silking, and high N transfer efficiency of BC treatment has the promotion of N transfer from vegetative organs to grains and increases grain N concentration (Xiao et al., 2017). Interestingly, under the treatments of C_2_, N uptake of the straw was higher than that of the grain, possibly due to that the source-sink incoordination caused by high BC treatment, which disturbed N transport to the grain (He et al., 2021). This leads to that the redundant nutrient was kept in such vegetative organs as stems and leaves, which causes the “luxury uptake” of nutrients (Travis et al., 2017). N application can enhance soil colloid adsorption and cation exchange capacity, reducing ammonium N loss (Duan et al., 2018). Proper N application is therefore useful for increasing the NRE of maize. However, when excessive N is added, N release is too rapid to match crop absorption and even substantially consume soil fertility (Xia et al., 2022). Furthermore, excessive fertilization not only significantly reduces N use efficiency, but also is unable to improve maize yield (Zhang et al., 2022; Nasar et al., 2023). The previous studies also indicate that it is an effective way for reasonable N application to improve fertilizer utilization efficiency, and N utilization efficiency initially increases and then decreases as N application increases (Miah et al., 2016).

### 4.4 Combining BC with N fertilizer improves grain quality

Appropriate N application is able to improve maize quality traits such as grain protein and oil content (Amanullah et al., 2009). In particular, grain protein concentration is continuously rises with increase in N application (Correndo et al., 2021). It is noted that both of low and high N applications have the impact on maize quality, which indicates that it is of great importance for adequate N application to raise maize quality (Hammad et al., 2011). Our study revealed that maize protein and oil content had a positive correlation with N application, but was negatively related to BC application. The reason may be that soil organic N is converted into inorganic N, which is then absorbed and utilized by crops (Sharifi et al., 2008). High C/N ratio of BC we used reduces the mineralization of organic N (Shen et al., 2021). Nitrogen is a crucial component required for the synthesis of cereal protein and oil (Zhang et al., 2020; Hafiz et al., 2023), and after plants absorb nitrogen, it is transported to the growth center organs in large quantities. The nitrogen stored in the vegetative organs during the early stage of growth begins to move outward shortly after flowering and is transported to the developing ear or grain (Ren et al., 2021). However, the results of this study found that high BC treatment led to N transfer from grain to straw, which also reduced the protein and oil content of maize. This is due to that BC competes with the organic-inorganic composite colloidal cations in the soil to adsorb nutrients which is also absorbed by crops, resulting in a decrease in protein content (Keiblinger et al., 2015). Previous studies have found a trade-off relationship between protein and starch content under given conditions (Butts-Wilmseyer et al., 2019; Singh et al., 2002), which is consistent with our findings. High N fertilizer promotes the increase of starch content in the early stage of grain filling, but causes crop premature senescence in the later period, which results in lower starch content for the high N fertilizer treatment than the normal treatment at harvest (Ma et al., 2012). The problem may be solved by the BC-N combination because the slow-release fertilizer effect of BC reduces the content of available N fertilizer in the soil in the early stage. It can also be seen from the results of our study that BC increases maize starch content. Soybean quality is generally characterized by protein and oil content. Its oil content is strongly affected by environmental conditions, but its variation trend is usually contrary to that of protein (Mertz-Henning et al., 2018). For instance, when BC increases from 0 to 8 t ha^-1^, soybean oil content shows an increased trend but protein content has the opposite one (Zahra et al., 2018), which is in accordance with our findings. One possible explanation for the result is that BC absorbs a large amount of nutrients in the prophase, which are then gradually released. The released nutrient rate may not coordinate with soybean protein formation but coordinate with fat formation (Liu et al., 2021). The other possible explanation for the result is that soybean protein and oil content may be mainly determined by the gene (Lin et al., 2022). In addition, soil microorganisms and environment also have an impact on crop quality (Studnicki et al., 2016; Kundel et al., 2020).

## 5 Conclusions

Appropriate BC and fertilizer application can have a positive impact on crop yield, N accumulation, WUE and NRE while also reducing ET. However, excessive BC can have negative effects on crop yield and quality by disturbing N translocation from grain to straw and reducing the benefits of intercropping. Based on our two-year experiment, we recommend a combination of 17.1-18.75 t ha^-1^ BC and 160.65-202.05 kg hac^-1^ N fertilizer to obtain high yields, water and N fertilizer use efficiency, and quality in the maize-soybean intercropping system. These findings have important implications for intercropping management and sustainable agricultural intensification.

## Data availability statement

The original contributions presented in the study are included in the article/**Supplementary Material**. Further inquiries can be directed to the corresponding authors.

## Author contributions

BY and IK conceptualized and wrote the main manuscript. BY, SL, and DL helped to perform lab analysis. IK and AR reviewed and edited the manuscript in the present form. JJ and LW helped with revisions and funding. All authors contributed to the article and approved the submitted version.

## References

[B1] Ahmed S. Raza M. A. Zhou T. Hussain S. Khalid M. H. B. Feng L.. (2018). Responses of soybean dry matter production, phosphorus accumulation, and seed yield to sowing time under relay intercropping with maize. Agronomy 8, 282. doi: 10.3390/agronomy8120282

[B2] Ajayi A. E. Horn R. (2016). Modification of chemical and hydro-physical properties of two texturally differentiated soils due to varying magnitudes of added biochar. Soil Tillage Res. 164, 34–44. doi: 10.1016/j.still.2016.01.011

[B3] Akhtar S. S. Guitong L. Mathias N. A. Fulai L. (2014). Biochar enhances yield and quality of tomato under reduced irrigation. Agric. Water Manage. 138, 37–44. doi: 10.1016/j.agwat.2014.02.016

[B4] Alkharabsheh H. M. Seleiman M. F. Battaglia L. B. Shami A. Jalal S. R. Bushra A. A.. (2021). Biochar and its broad impacts in soil quality and Fertility,Nutrient leaching and crop productivity: a review. Agronomy 11, 5. doi: 10.3390/agronomy11050993

[B5] Amanullah Khattak R. A. Khalil S. K. (2009). Plant density and nitrogen effects on maize phenologyand grain yield. J. Plant Nutr. 32 (2), 246–260. doi: 10.1080/01904160802592714

[B6] Butts-Wilmsmeyer C. J. Seebauer J. R. Singleton L. Below F. E. (2019). Weather during key growth stages explains grain quality and yield of maize. Agronomy. 9, 16.

[B7] Castellini M. Giglio L. Niedda M. Palumbo A. D. Ventrella D. (2015). Impact of biochar addition on the physical and hydraulic properties of a clay soil. Soil Tillage Res. 154, 1–13. doi: 10.1016/j.still.2015.06.016

[B8] Chen L. Chen Q. C. Rao P. H. Yan L. L. Shakib A. Shen G. Q. (2018). Formulating and optimizing a novel biochar-based fertilizer for simultaneous slow-release of nitrogen and immobilization of cadmium. Sustainability 10, 8. doi: 10.3390/su10082740

[B9] Chen Y. Shinogi Y. Taira M. (2010). Influence of biochar use on sugarcane growth, soil parameters, and groundwater quality. Aust. J. Soil Res. 48, 526–530. doi: 10.1071/SR10011

[B10] Correndo A. A. Fernandez J. A. Vara P. V. Ciampitti I. A. (2021). Do water and nitrogenmanagement practices impact grain quality in maize? Agronomy 11 (9), 1851. doi: 10.3390/agronomy11091851

[B11] Duan R. Long X. E. Tang Y. F. Wen J. Su S. M. Bai L. Y.. (2018). Effects of different fertilizer application methods on the community of nitrifiers and denitrifiers in a paddy soil. J. Soils Sediments 18 (1), 24–38. doi: 10.1007/s11368-017-1738-9

[B12] Duchene O. Vian J.-F. Celette F. (2017). Intercropping with legume for agroecological cropping systems: complementarity andfacilitation processes and the importance of soil microorganisms. a review. Agric. Ecosyst. Environ. 240, 148–161. doi: 10.1016/j.agee.2017.02.019

[B13] Faloye O. T. Alatise M. O. Ajayi A. E. Ewulo B. S. (2017). Synergistic effects of biochar and inorganic fertiliser on maize yield in an alfisol under drip irrigation. Soil Tillage Res. 174, 214–220. doi: 10.1016/j.still.2017.07.013

[B14] Faloye O. T. Alatise M. O. Ajayi A. E. Ewulo B. S. (2019). Effects of biochar and inorganic fertilizer applications on growth, yield and water use efficiency of maize under deficit irrigation. Agric. Water Manage. 217, 165–178. doi: 10.1016/j.agwat.2019.02.044

[B15] Farhangi-Abriz S. Torabian S. Qin R. J. Noulas C. Lu Y. Y. Gao S. D. (2021). Biochar effects on yield of cereal and legume crops using meta-analysis. Sci. Total Environ. 775, 145869. doi: 10.1016/j.scitotenv.2021.145869

[B16] Feng L. Raza M. A. Chen Y. Khalid M. H. B. Meraj T. A. Ahsan F.. (2019). Narrow-wide row planting pattern improves the light environment and seed yields of intercrop species in relay intercropping system. PloS One 14, 2. doi: 10.1371/journal.pone.0212885 PMC6391028 30807607

[B17] Guo L. L. Yu H. W. Kharbach M. Wang J. W. (2021). The response of nutrient uptake, photosynthesis and yield of tomato to biochar addition under reduced nitrogen application. Agronomy 11, 8. doi: 10.3390/agronomy11081598

[B18] Hafiz M. H. Abbas F. Ashfaq A. Wajid F. Carol J. W. Gerrit H. (2023). Water and nitrogen management influence on oil and protein concentration in maize. Agron. J. 2023, 557–568. doi: 10.1002/agj2.21275

[B19] Hammad H. M. Ahmad A. Khaliq T. Farhad W. Mubeen M. (2011). Optimizing rate of nitrogen application for higher yield and quality in maize under semiarid environment. Crop Environ. 2 (1), 38–41. https://www.researchgate.net/publication/268422853

[B20] He Z. H. Hong T. T. Cai Z. L. Yang Z. Li M. Zhang Z. (2021a). Determination of amount of irrigation and nitrogen for comprehensive growth of greenhouse cucumber based on multi-level fuzzy evaluation. Int. J. Agric. Biol. Eng. 14 (2), 35–42. doi: 10.25165/j.ijabe.20211402.5785

[B21] He Z. H. Li M. Cai Z. L. Zhao R. S. Hong T. T. Yang Z.. (2021b). Optimal irrigation and fertilizer amounts based on multi-level fuzzy comprehensive evaluation of yield, growth and fruit quality on cherry tomato. Agric. Water Manage. 243, 1–10. doi: 10.1016/j.agwat.2020.106360

[B22] He D. W. Zhao. Y. Z. Gao J. P. Sui Y. H. Xin W. Yi J.. (2021). Effects of biochar application combined with nitrogen fertilizer on yield formation of japonica rice and the immediate and residual effects of nitrogen. J. Plant Nutr. Fertilizers 27 (12), 2114–2124. doi: 10.11674/zwyf.2021244

[B23] Hou M. M. Lin Z. Y. Chen J. N. Zhai Y. M. Jin Q. Zhong F. L. (2018). Optimization on theburied depth of subsurface drainage under greenhouse condition based on entropy evaluation method. Entropy 20 (11), 10. doi: 10.3390/e20110859 PMC7512420 33266583

[B24] Ibrahim M. M. Tong C. X. Hu K. Zhou B. Q. Xing S. H. Mao Y. L. (2020). Biochar-fertilizer interaction modifies n-sorption, enzyme activities and microbial functional abundance regulating nitrogen retention in rhizosphere soil. Sci. Total Environmen 739, 140065. doi: 10.1016/j.scitotenv.2020.140065 32758953

[B25] Iqbal N. Hussain S. Ahmed Z. Yang F. Wang X. Liu W.. (2019). Comparative analysis of maize–soybean strip intercropping systems: a review. Plant Prod. Sci. 22, 131–142. doi: 10.1080/1343943X.2018.1541137

[B26] Jeffery S. Meinders B. J. M. Stoof R. C. Bezemer T. M. Tess F. J. Mommer L.. (2015). Biochar application does not improve the soil hydrological function of a sandy soil. Geoderma 251/252, 47–54. doi: 10.1016/j.geoderma.2015.03.022

[B27] Jeffery S. Verheijen F. G. A. vanderVelde M. Bastos A. C. (2011). A quantitative review of the effects of biochar application to soils on crop productivity using meta-analysis. Agr. Ecosyst. Environ. 144, 175–187. doi: 10.1016/j.agee.2011.08.015

[B28] Jin Z. E. Chen C. Chen X. M. Jiang F. Hopkins I. Zhang X. L.. (2019). Soil acidity, available phosphorus content, and optimal biochar and nitrogen ertilizer application rates: a five-year field trial in upland red soil, China. Field Crops Res. 232, 77–87. doi: 10.1016/j.fcr.2018.12.013

[B29] Jindo K. Audette Y. Higashikawa F. S. Silva C. A. Akashi K. Mastrolonardo G.. (2020). Role of biochar in promoting circular economy in the agriculture sector. part 1: a review of the biochar roles in soil n, p and K cycles. Chem. Biol. Technol. Agric. 7, 15. doi: 10.1186/s40538-020-00182-8

[B30] Kammann C. I. Linsel S. Gößling J. W. Koyro H. W. (2011). Influence of biochar on drought tolerance of chenopodiumquinoa willd and on soil-plant relations. Plant Soil 345, 195–210. doi: 10.1007/s11104-011-0771-5

[B31] Keiblinger K. M. Liu D. Mentler A. Zehetner F. Zechmeister-Boltenstern S. (2015). Biochar application reduces protein sorption in soil. Organic Geochemistry 87, 21–24. doi: 10.1016/j.orggeochem.2015.06.005

[B32] Kundel D. Bodenhausen N. Jørgensen H. B. Truu J. Birkhofer K. Hedlund K.. (2020). A. effects of simulated drought on biological soil quality, microbial diversity and yields under long-term conventional and organic agriculture. FEMS Microbiol. Ecol. 96, 12. doi: 10.1093/femsec/fiaa205 PMC7705324 33016314

[B33] Kundu S. Khare D. Mondal A. (2017). Landuse change impact on sub-watersheds prioritization by analytical hierarchy process (AHP). Ecol. Inf. 42, 100–113. doi: 10.1016/j.ecoinf.2017.10.007

[B34] Lee E. A. Tollenaar M. (2007). Physiological basis of successful breeding strategies for maize grain yield. Crop Sci. 47, S-202–S-215. doi: 10.2135/cropsci2007.04.0010IPBS

[B35] Li B. Wu H. M. Zhang F. F. Li C. J. Li X. X. Lambers H.. (2016). Root exudates drive interspecific facilitation by enhancing nodulation and N2 fixation. Proc. Natl. Acad. Sci. United States America 113 (23), 6496–6501. doi: 10.1073/pnas.1523580113 PMC4988560 27217575

[B36] Lin S. F. Pi Y. J. Long D. Y. Duan J. J. Zhu X. T. Wang X. L.. (2022). Impact of organic and chemical nitrogen fertilizers on the crop yield and fertilizer use efficiency of soybean–maize intercropping systems. Agriculture 12 (9). doi: 10.3390/agriculture12091428

[B37] Liu B. J. Chen X. H. Lian. Y. Q. Wu L. L. (2013). Entropy-based assessment and zoning of rainfall distribution. J. Hydrology 490, 32–40. doi: 10.1016/j.jhydrol.2013.03.020

[B38] Luan C. He W. Su X. Wang X. M. Bai Y. K. Wang L. X. (2021). Effects of biochar on soil water and temperature, nutrients, and yield of maize/soybean and maize/peanut intercropping systems. Int.Agroghys 35, 365–373. doi: 10.31545/intagr/144133

[B39] Luo K. Yuan X. T. Xie C. Liu S. S. Chen P. Du Q.. (2022). Diethyl aminoethyl hexanoate increase relay strip intercropping soybean grain by optimizing photosynthesis aera and delaying leaf senescence. Fontiers Plant Sci. 12. doi: 10.3389/fpls.2021.818327 PMC8767051 35069671

[B40] Ma R. X. Wang J. S. Li X. Z. Liu W. C. (2012). Effect of different NPK fertilizers cooperating application on yield and quality of high starch maize. Appl. Mechanics Materials 214, 423–429. doi: 10.4028/www.scientific.net/AMM.214.423

[B41] Mertz-Henning L. M. Ferreira L. C. Henning F. A. J. Mandarino M. G. Santos E. D. Oliveira M. C. N. D.. (2018). Effect of water deficit-induced at vegetative and reproductive stages on protein and oil content in soybean grains. Agron. J. 8, 3. doi: 10.3390/agronomy8010003

[B42] Miah M. Gaihre Y. K. Hunter G. Singh U. Hossain S. A. (2016). Fertilizer deep placement increases rice production: evidence from ‘farmers’ fields in southern Bangladesh. Agron. J. 108 (2), 805–812. doi: 10.2134/agronj2015.0170

[B43] Mohammad G. Petr K. Reinhard W. N. Marek K. Elnaz A. Daniel B.. (2022). Interaction of biochar with chemical, green and biological nitrogen fertilizers on nitrogen use efficiency indices. Agronomy 12, 9. doi: 10.3390/agronomy12092106

[B44] Mugi-Ngenga E. Bastiaans L. Zingore S. Anten N. P. R. Giller K. E. (2022). The role of nitrogen fixation and crop n dynamics on performance and legacy effects of maize-grain legumes intercrops on smallholder farms in Tanzania. Eur. J. Agron. 141. doi: 10.1016/j.eja.2022.126617

[B45] Nasar J. Zhai J. C. Khan R. Gul H. Gitari H. Shao Z. Q.. (2023). Maize-soybean intercropping at optimal n fertilization increases the n uptake, n yield and n use efficiency of maize crop by regulating the n assimilatory enzymes. Front. Plant Sci., 13. doi: 10.3389/fpls.2022.1077948 PMC9846272 36684768

[B46] Omara P. Aula L. Oyebiyi F. B. Eickhoff E. M. Carpenter J. Raun W. R. (2020). Biochar application in combination with inorganic nitrogen improves maize grain yield, nitrogen uptake, and use efficiency in temperate soils. Agronomy 10 (9). doi: 10.3390/agronomy10091241

[B47] Purakayastha T. J. Bera T. Bhaduri D. Sarkar B. Mandal S. Wade P.. (2019). A review on biochar modulated soil condition improvements and nutrient dynamics concerning crop yields: pathways to climate change mitigation and global food security. Chemosphere 227, 345–365. doi: 10.1016/j.chemosphere.2019.03.170 30999175

[B48] Raza M. A. Feng L. Y. Iqbal N. Ahmed M. Chen Y. K. Bin Khalid M. H.. (2019b). Growth and development of soybean under changing light environments in relay intercropping system. Peerj 7, e7262. doi: 10.7717/peerj.7262 31372317 PMC6659667

[B49] Raza M. A. Feng L. Y. van der Werf W. Cai G. R. Khalid M. H. B. Iqbal N.. (2019a). Narrow-wide-row planting pattern increases the radiation use efficiency and seed yieldof intercrop species in relay-intercropping system. Food Energy Secur 8. doi: 10.1002/fes3.170 PMC6391028 30807607

[B50] Raza M. A. Feng L. Y. van der Werf W. Iqbal N. Khan I. Hassam M. J.. (2019c). Optimum leaf defoliation: a new agronomic approach for increasing nutrient uptake and land equivalent ratio of maize soybean relay intercropping system. Field Crops Res. 244. doi: 10.1016/j.fcr.2019.107647

[B51] Raza M. A. Yasin H. S. Gul H. Qin R. Din A. M. U. Khalid M. H. B.. (2022). Maize/soybean strip intercropping produces higher crop yields and saves water under semi-arid conditions. Front. Plant Sci. 13. doi: 10.3389/fpls.2022.1006720 PMC9667818 36407615

[B52] Ren H. Jiang Y. Zhao M. Qi H. Li C. F. (2021). Nitrogen supply regulates vascular bundle structure and matter transport characteristics of spring maize under high plant density. Front. Plant Sci. 11. doi: 10.3389/fpls.2020.602739 PMC7820718 33488648

[B53] Rogovska N. Laird D. A. Rathke S. J. Karlen D. L. (2014). Biochar impact on Midwestern mollisols and maize nutrient availability. Geoderma 230-231, 340–347. doi: 10.1016/j.geoderma.2014.04.009

[B54] Sahoo S. Sil I. Dhar A. Debsarkar A. Das P. Kar A. (2018). Future scenarios of land-use suitability modeling for agricultural sustainability in a river basin. J. Cleaner Production 205, 313–328. doi: 10.1016/j.jclepro.2018.09.099

[B55] Seleiman M. F. Alotaibi M. A. Alhammad B. A. Alharbi B. M. Refay Y. Badawy S. A. (2020). Effects of ZnO nanoparticles and biochar of rice straw and cow manure on characteristics of contaminated soil and sunflower productivity, oil quality, and heavy metals uptake. Agronomy 10, 790. doi: 10.3390/agronomy10060790

[B56] Sharifi M. Zebarth B. J. Burton D. L. Grant C. A. Porter G. A. (2008). Organic amendment history and crop rotation effects on soil nitrogen mineralization potential and soil nitrogen supply in a potato cropping system. Agron. J. 100, 1562–1572. doi: 10.2134/agronj2008.0053

[B57] Shen S. Z. Wan C. Ma X. J. Hu Y. K. Wang F. Zhang K. Q. (2021). Nitrogen mineralization characteristics of organic fertilizer in livestock and poultry under the condition of flood-drought rotation. J. Agric. Environ. Sci. 40, 2513–2520. doi: 10.11654/jaes.2021-1032

[B58] Singh M. Paulsen M. R. Tian L. Yao H. (2002). Site-Specific Study of Corn Protein, Oil, and Extractable Starch Variability Using NIT Spectroscopy, ASAE Meeting Paper, 02–1111. ASAE: St. Joseph, MI, USA.

[B59] Solaiman Z. M. Shafi M. I. Beamont E. Anawar H. M. (2020). Poultry litter biochar increases mycorrhizal colonization, soil fertility and cucumber yield in a fertigation system on sandy soil. Agriculture 10, 480. doi: 10.3390/agriculture10100480

[B60] Studnicki M. Wijata M. Sobczy’ nski G. Samborski S. Gozdowski D. Rozbicki J. (2016). Effect of genotype, environment and crop management on yield and quality traits in spring wheat. J. Cereal Sci. 72, 30–37. doi: 10.1016/j.jcs.2016.09.012

[B61] Sun H. J. Zhang H. C. Shi W. M. Zhou M. Y. Ma X. F. (2019). Effect of biochar on nitrogen use efficiency, grain yield and amino acid content of wheat cultivated on saline soil. Plant Soil Environ. 65, 83–89. doi: 10.17221/525/2018-PSE

[B62] Travis G. Jacob N. Greg S. Bill D. Manish. R. (2017). Mid-season leaf glutamine predicts end-season maize grain yield and nitrogen content in response to nitrogen fertilization under field conditions. Agronomy 7, 2. doi: 10.3390/agronomy7020041

[B63] Wang D. Jiang P. Zhang H. Yuan W. (2020). Biochar production and applications in agro and forestry systems: a review. Sci. Total Environ. 723, 137775. doi: 10.1016/j.scitotenv.2020.137775 32213399

[B64] Wang Q. S. Yuan X. L. Zhang J. Gao Y. Hong J. L. Zuo J. (2015). Assessment of the sustainable development capacity with the entropy weight coefficient method. Sustainability 7 (10), 13542–13563. doi: 10.3390/su71013542

[B65] Weber K. Quicker P. (2018). Properties of biochar. Fuel 217, 240–261. doi: 10.1016/j.fuel.2017.12.054

[B66] Wu Y. S. Gong W. Z. Yang F. Wang X. C. Yong T. W. Yang W. Y. (2016). Responses to shade and subsequent recovery of soya bean in maize-soya bean relay strip intercropping. Plant Production Sci. 19 (2), 206–214. doi: 10.1080/1343943X.2015.1128095

[B67] Wu W. A. Han J. Y. Gu Y. N. Li T. Xu X. R. Jiang Y. H.. (2022). Impact of biochar amendment on soil hydrological properties and crop water use efficiency: a global meta-analysis and structural equation model. Global Change Biol. Bioenergy 14 (6), 657–668. doi: 10.1111/gcbb.12933

[B68] Xia H. Riaz M. Zhang M. Y. Liu B. Li Y. X. El-Desouki Z.. (2022). Biochar-n fertilizer interaction increases n utilization efficiency by modifying soil C/N component under n fertilizer deep placement modes. Chemospher 286 (1). doi: 10.1016/j.chemosphere.2021.131594 34346321

[B69] Xiao Q. Zhu L. X. Shen Y. F. Li S. Q. (2016b). Sensitivity of soil water retention and availability to biochar addition in rainfed semiarid farmland during a three-year field experiment. Field Crop Res. 196, 284–293. doi: 10.1016/j.fcr.2016.07.014

[B70] Xiao Q. Zhu L. X. Tang L. Shen Y. F. Li S. Q. (2017). Responses of crop nitrogen partitioning, translocation and soil nitrogen residue to biochar addition in a temperate dryland agricultural soil. Plant Soil 418 (1-2), 405–421. doi: 10.1007/s11104-017-3304-z

[B71] Xiao Q. Zhu L. X. Zhang H. P. Li X. Y. Shen Y. F. Li S. Q. (2016a). Soil amendment with biochar increases maize yields in a semiarid region by improving soil quality and root growth. Crop Pasture Sci. 67 (5), 495–507. doi: 10.1071/CP15351

[B72] Xu Z. Li C. J. Zhang C. C. Yu Y. van der Werf W. Zhang F. S. (2020). Intercropping maize and soybean increases efficiency of land and fertilizer nitrogen use, a meta-analysis. Field Crops Res. 246. doi: 10.1016/j.fcr.2019.107661

[B73] Yang B. B. Xu K. Zhang Z. (2020). Mitigating evaporation and desiccation cracks in soil with the sustainable material biochar. Soil Sci. Soc. America J. 84(2), 461–471. doi: 10.1002/saj2.20047

[B74] Yao Q. Liu J. J. Yu Z. H. Li Y. S. Jin J. Liu X. B.. (2017). Three years of biochar amendment alters soil physiochemical properties and fungal community composition in a black soil of northeast China. Soil Biol. Biochem. 110, 56–67. doi: 10.1016/j.soilbio.2017.03.005

[B75] Zahra A. Hasan E. Bahman G. Abolfazl F. (2018). Effects of biochar and bio-fertilizer on yield and qualitative properties of soybean and some chemical properties of soil. Arabian J. Geosciences 11, 672. doi: 10.1007/s12517-018-4041-1

[B76] Zhang L. Liang Z. Y. He X. M. Meng Q. F. Hu Y. C. Schmidhalter U.. (2020). Improving grain yield and protein concentration of maize (Zea mays l.) simultaneously by appropriate hybrid selection and nitrogen management. Field Crops Res. 249. doi: 10.1016/j.fcr.2020.107754

[B77] Zhang F. S. Wang J. Q. Zhang W. F. Cui Z. L. Ma W. Q. Chen X. P.. (2008). Nutrient use efficiencies of major cereal crops in China and measures for improvement. Acta Pedol. Sin. 45, 915–924.

[B78] Zhang Y. J. Ye C. Su Y. W. Peng W. C. Lu R. Liu Y. X.. (2022). Soil acidification caused by excessive application of nitrogen fertilizer aggravates soil-borne diseases: evidence from literature review and field trials. Agriculture Ecosyst. Environ. 340. doi: 10.1016/j.agee.2022.108176

[B79] Zhong F. Hou M. He B. Chen I. (2017). Assessment on the coupling effects of drip irrigation and organic fertilization based on entropy weight coefficient model. Peer J. 5, e3855. doi: 10.7717/peerj.3855 29018603 PMC5629958

[B80] Zhou M. H. Butterbach-Bahl K. (2014). Assessment of nitrate leaching loss on a yield-scaled basis from maize and wheat croppingsystems. Plant Soil 374, 977–991. doi: 10.1007/s11104-013-1876-9

