# Supplementary material for: Assessing the impact of biochar and nitrogen application on yield, water-nitrogen use efficiency and quality of intercropped maize and soybean
Source: Front Plant Sci. 2023 May 8;14:1171547. doi: 10.3389/fpls.2023.1171547 (PMC10200913; doi:10.3389/fpls.2023.1171547)
Supplement: Supplementary file 1 [file Table_1.docx]

**Supplementary Table 1** Subjective weights of factors based on the AHP method

|  | Index | Local weights | Final weight | Consistency test parameter | | |
| --- | --- | --- | --- | --- | --- | --- |
|  | *u*_1_ | 0.54 | 0.54 | C_R_=0.0088＜0.1 | | |
| *U*~*u*_i_ | *u*_2_ | 0.297 | 0.297 | λ_max_=3.0092 | | |
|  | *u*_3_ | 0.163 | 0.163 |  |  |  |
| *u*_1_~ *u*_1j_ | *u*_11_ | 0.75 | 0.405 | C_R_=0＜0.10 | | |
|  | *u*_12_ | 0.25 | 0.135 | λ_max_=2 | | |
| *u*_2_~ *u*_2j_ | *u*_21_ | 0.5 | 0.148 | C_R_=0＜0.10 | | |
|  | *u*_21_ | 0.5 | 0.148 | λ_max_=2 | | |
| *u*_3_~ *u*_3j_ | *u*_31_ | 0.068 | 0.011 | C_R_=0.0888＜0.1 | | |
|  | *u*_32_ | 0.419 | 0.068 |  |  |  |
|  | *u*_33_ | 0.178 | 0.029 | λ_max_=5.3942 | | |
|  | *u*_34_ | 0.1 | 0.016 |  |  |  |
|  | *u*_35_ | 0.235 | 0.038 |  |  |  |

*When C_R_<0.1, the consistency test is passed and the indicatorevaluation matrix is accepted.*

**Supplementary Table 2**. Objective weights of sub-factors based on the entropy method

| 2021 | | | 2022 | | | | |
| --- | --- | --- | --- | --- | --- | --- | --- |
| Index | Value information entropy (e) | Information utility value (d) | | Weight | Value information entropy (e) | Information utility value (d) | Weight |
| *u*_11_ | 0.877 | 0.123 | | 0.101 | 0.908 | 0.092 | 0.097 |
| *u*_12_ | 0.923 | 0.077 | | 0.063 | 0.91 | 0.09 | 0.102 |
| *u*_21_ | 0.806 | 0.194 | | 0.161 | 0.885 | 0.115 | 0.114 |
| *u*_21_ | 0.879 | 0.121 | | 0.1 | 0.903 | 0.097 | 0.102 |
| *u*_31_ | 0.922 | 0.078 | | 0.064 | 0.905 | 0.095 | 0.10 |
| *u*_32_ | 0.804 | 0.196 | | 0.162 | 0.931 | 0.069 | 0.073 |
| *u*_33_ | 0.905 | 0.095 | | 0.079 | 0.815 | 0.185 | 0.196 |
| *u*_34_ | 0.774 | 0.226 | | 0.187 | 0.9 | 0.1 | 0.106 |
| *u*_35_ | 0.9 | 0.1 | | 0.083 | 0.894 | 0.106 | 0.112 |

**Supplementary Table3** Weight of single indicator based on the game theory

| Index |  | *u*_11_ | *u*_12_ | *u*_21_ | *u*_21_ | *u*_31_ | *u*_32_ | *u*_33_ | *u*_34_ | *u*_35_ |
| --- | --- | --- | --- | --- | --- | --- | --- | --- | --- | --- |
| Combination | 2021 | 0.294 | 0.104 | 0.136 | 0.122 | 0.022 | 0.063 | 0.056 | 0.030 | 0.068 |
| weight | 2022 | 0.336 | 0.126 | 0.142 | 0.138 | 0.030 | 0.069 | 0.065 | 0.036 | 0.054 |
